# Beyond Global IQ: Identifying Subgroups of Intellectual Functioning in Dyslexia Through Latent Profile Analysis

**DOI:** 10.3390/jintelligence13110144

**Published:** 2025-11-10

**Authors:** Bartosz M. Radtke, Ariadna Łada-Maśko, Paweł Jurek, Michał Olech, Shally Novita, Urszula Sajewicz-Radtke

**Affiliations:** 1 Laboratory of Psychological and Educational Tests, Ul. Czarnieckiego 5A/1, 80-239 Gdańsk, Poland; radtke@pracowniatestow.pl; 2 Institute of Psychology, University of Gdansk, Ul. Bażyńskiego 8, 80-309 Gdańsk, Poland; ariadna.lada@ug.edu.pl (A.Ł.-M.); pawel.jurek@ug.edu.pl (P.J.); 3 Department of Psychology, Medical University of Gdańsk, Ul. M. Skłodowskiej-Curie 3a, 80-210 Gdańsk, Poland; michal.olech@gumed.edu.pl; 4 Center for Psychological Innovation and Research, Faculty of Psychology, Universitas Padjadjaran, Jl. Raya Sumedang Jatinangor KM 21, Jatinangor 45363, West Java, Indonesia; s.novita@unpad.ac.id

**Keywords:** cognitive heterogeneity, cognitive profiles, latent variable modeling, SB5 assessment, developmental dyslexia, reading difficulties, learning difficulties, neurodevelopmental disorder, clinical and educational applications

## Abstract

Dyslexia, a heterogeneous neurodevelopmental disorder, is characterized by persistent reading and spelling difficulties despite average intellectual potential. Although intellectual functioning in dyslexia is often described as average, emerging evidence suggests meaningful within-group variability. This study examined whether children and adolescents with dyslexia exhibited distinct intellectual profiles based on the Stanford-Binet Intelligence Scales, Fifth Edition (SB5). Data were obtained from a large, diagnostically verified sample of 3458 individuals aged 10–19 years assessed in psychological-pedagogical counseling centers across Poland. We used latent profile analysis (LPA) of all 10 SB5 subtests and compared models that specified 2–6 latent classes. The optimal solution identified two profiles: (a) a small subgroup (5%) with globally reduced intellectual functioning and a profound deficit in verbal working memory (>3 standard deviations below the norm) and (b) the predominant subgroup (95%) with broadly average intellectual functioning and relatively preserved reasoning abilities. Profile membership was associated with socioeconomic status; the low-functioning subgroup was associated with lower parental education and age, as younger participants were more likely to belong to this group. These findings highlight the dimensional nature of intellectual heterogeneity in dyslexia and underscore the diagnostic value of profile-based approaches over global intelligence quotient (IQ) scores.

## 1. Introduction

Dyslexia is defined as persistent difficulties in accurate and/or fluent word recognition and spelling not attributed to inadequate instruction or general intellectual disability (Odegard et al. 2024). Despite this shared behavioral phenotype, dyslexia is highly heterogeneous at both the cognitive and neurobiological levels, which poses challenges for theory and assessment (Giofrè et al. 2019; Pacheco et al. 2014; Zoubrinetzky et al. 2014). Two implications follow. Theoretically, multiple-deficit models imply mixtures of risk constellations rather than a single canonical profile; group averages can mislead mechanism-level inference (Dębska et al. 2022; Hall and Vaughn 2021). For assessment, aggregation across heterogeneous profiles can attenuate or even invert clinically meaningful differences (Simpson-type masking); profile-level interpretation should supplement global indices (Bonovas and Piovani 2023). We adopt this person-centered perspective to align inference with documented heterogeneity. Although individuals with dyslexia often display overlapping deficits, evidence for distinct cognitive subtypes remains mixed, suggesting a dimensional rather than categorical organization (Dębska et al. 2022). Nevertheless, findings from cognitive-clustering and neuroimaging studies suggest meaningful within-group differentiation. Cluster-analysis studies have identified distinct profiles according to different constellations of domain-specific deficits, which support the multiple-deficit models of dyslexia. Neuroimaging studies, including voxel-based morphometry, have revealed subtype-specific neuroanatomical signatures, with structural variations observed in the left inferior frontal gyrus, cerebellum, putamen, and parietal cortex (Chalmpe and Vlachos 2024; Jednoróg et al. 2014). Recent electrophysiological and neuroimaging overviews further emphasize distributed, and partly non-overlapping, alterations within reading and language networks, consistent with cognitive heterogeneity (Cainelli et al. 2023). Furthermore, prevalence studies have increasingly conceptualized dyslexia as a severity-graded, distributional phenomenon, which reinforces the expectation of intra-group diversity (Wagner et al. 2020).

The Cattell–Horn–Carroll (CHC) framework provides a well-validated taxonomy for organizing cognitive abilities into broad intellectual abilities (McGrew 2023; McGrew et al. 2023). Recent psychometric network analyses support the major CHC broad factors and underscore the centrality of working memory and attentional control. However, they caution that reliance on global g (general intelligence) may obscure clinically meaningful variation. Evidence further demonstrates the generalizability of CHC-based indices, supporting their use for comparative profiling across diverse populations (McGrew et al. 2023; Wilson et al. 2023).

At the group level, studies on intellectual functioning in dyslexia consistently revealed a characteristic pattern: relatively weak working memory and processing speed, accompanied by comparatively preserved verbal and non-verbal reasoning (Giofrè et al. 2019; Poletti 2016). Hence, although overall intellectual functioning typically falls within the average range, the internal organization of cognitive abilities diverges from that of peers with typical development. However, Abu-Hamour and Al Hmouz (2020) caution against overgeneralization and note that intellectual profiles in dyslexia are not uniform but rather comprise heterogeneous constellations of strengths and weaknesses. This heterogeneity motivates analyses that minimize bias from aggregation and emphasize class- or profile-specific inference (Kulesz et al. 2024).

Large-scale investigations have demonstrated both commonalities and subgroup differentiation. Toffalini et al. (2017) found that although deficits in working memory and processing speed were shared across Specific Learning Disorder (SLD) categories, cognitive profiles diverged in strengths and weaknesses. Similarly, Wechsler Intelligence Scale for Children, Fourth Edition (WISC-IV; Wechsler 2003) analyses revealed that discrepancies between reasoning (Verbal Comprehension, Perceptual Reasoning) and efficiency indices (Working Memory, Processing Speed) may serve as cognitive markers of dyslexia, although substantial within-group variability persists (Giofrè et al. 2019; Poletti 2016). These findings emphasize the importance of examining intellectual functioning at the profile level rather than relying exclusively on global intelligence quotient (IQ) scores (Fiorello et al. 2007).

Most literature on intellectual profiling in neurodevelopmental disorders has relied on the WISC. However, whether WISC indices reflect psychometric artifacts rather than coherent cognitive constructs remains a concern, which limits their utility for profile-based analyses (Dombrowski et al. 2015). Conversely, the Stanford-Binet Intelligence Scales, Fifth Edition (SB5), is considered better suited when a detailed and comprehensive clinical assessment is required. Like most contemporary intelligence measures, the SB5 is grounded in the CHC framework; however, it encompasses the broadest range of cognitive domains by integrating both verbal and nonverbal tasks and thus provides the most extensive coverage of intellectual abilities (Flanagan et al. 2022). Importantly, unlike the WISC, the SB5 does not include speeded subtests, which may be particularly advantageous for individuals with dyslexia, for whom time pressure can obscure true ability levels (Wilson and Gilmore 2012). Furthermore, evidence indicates that it can reveal distinct constellations of strengths and weaknesses in children with learning disorders (Tippin 2007), which underscores its suitability for examining intellectual heterogeneity in dyslexia. In line with current identification guidance, we view profile-based interpretation as complementary to, not a replacement for, established diagnostic protocols (Hall and Vaughn 2021).

To our best knowledge, this study is the largest investigation to employ latent profile analysis (LPA) of the full SB5 battery (Roid et al. 2017b) in a diagnostically confirmed sample of individuals with dyslexia. Previous LPA studies have focused on reading, language, or memory measures rather than a full intelligence scale (Capin et al. 2021; Foorman et al. 2017; Gray et al. 2019; Holopainen et al. 2020). Notably, this study uses all 10 standardized SB5 subscales rather than relying solely on global IQ. Prior research has often emphasized general intelligence or selected subtests, which may obscure meaningful intra-individual variability. This study incorporates a multidimensional profile of both verbal and non-verbal abilities, including fluid reasoning, knowledge, quantitative reasoning, visual-spatial processing and working memory, and captures the nuanced cognitive architecture underlying intellectual functioning in dyslexia (Olech et al. 2024). This fine-grained approach is important as individuals with dyslexia often exhibit significant intra-individual differences across domains, despite assumptions of average intellectual functioning (Abu-Hamour and Al Hmouz 2020; Dębska et al. 2022; Giofrè et al. 2019; Toffalini et al. 2017). Examining within-IQ variations deepens our understanding of heterogeneity in dyslexia and challenges simplified interpretations of the dyslexia-intelligence relationship.

Primarily, this study aimed to address whether individuals with dyslexia exhibited one or more distinct profiles of intellectual functioning (RQ1). Based on prior research, we hypothesized that dyslexia would not be associated with a cognitively homogeneous profile (Capin et al. 2021; Gray et al. 2019; Zoubrinetzky et al. 2014). Instead, we anticipated identifying at least two distinct profiles characterized by different constellations of cognitive strengths and weaknesses. Furthermore, we examined whether these profiles varied according to age, gender, or parental socioeconomic status (RQ2). Identifying demographic correlates of cognitive profiles may shed light on the developmental and contextual factors that shape cognitive diversity in dyslexia (Giofrè et al. 2019; Rakesh et al. 2025). The second aim was exploratory; we sought to describe associations that may guide future hypothesis-driven research rather than assess specific hypotheses.

Addressing these questions requires a large sample and multidimensional assessment of intellectual functioning. Small-scale studies or simple mean comparisons are insufficient for detecting subtle but meaningful differences. To ensure statistical power and diagnostic precision, we utilized a unique, large-scale dataset that comprised numerous children and adolescents diagnosed with dyslexia who received psychological and educational support from psychological-pedagogical counseling centers across Poland. We applied LPA to this diagnostically verified and nationally representative sample, comprehensively assessed via the SB5, and provide a methodologically rigorous contribution to the understanding of cognitive heterogeneity in dyslexia.

## 2. Materials and Methods

### 2.1. Participants and Procedure

Data from 3458 children and adolescents, aged 10–19 years (M = 13.13, SD = 1.94), diagnosed with dyslexia were analysed. These data were obtained from a larger, publicly available database on intelligence assessment in children and youth receiving psychological and educational support in Poland (see Olech et al. 2024). Diagnoses of specific learning disorder with impairment in reading (dyslexia) were established prior to research enrolment by psychological-pedagogical counselling centers (PPCSs) following national procedures. While core diagnostic steps are standardized at the national level, minor variation in the selection of validated literacy instruments may occur across centers. All participants in the present study had a PPCS-confirmed diagnosis at the time of data entry into the database. Table 1 presents the demographic characteristics, including age, gender distribution, and parental education. The study followed ethical guidelines; informed consent was obtained from the parents/guardians of all participants.

### 2.2. Subsection

The SB5 (Roid et al. 2017b), an assessment of intellectual functioning designed for individuals aged ≥ 2 years, comprises 10 subtests that generate a Full Scale IQ (FSIQ). The Stanford–Binet Intelligence Scales, Fifth Edition (SB5), index general ability across five broad factors: Fluid Reasoning (FR; reasoning with novel information), Knowledge (KN; breadth of acquired information and vocabulary), Quantitative Reasoning (QR; number concepts and arithmetic problem solving), Visual–Spatial Processing (VS; analysis and manipulation of visual–spatial relations), and Working Memory (WM; temporary storage and mental manipulation of information). Each factor is assessed with both nonverbal and verbal indicators, and scores are age-normed following manual conventions. The SB5 was normalized on a country-wide representative sample of 3246 individuals. The results were characterized by high reliability for the full scale and non-verbal IQ, verbal IQ, and short versions (0.92–0.98). The average reliability of the five and 10 factors was 0.88–0.91 and 0.78–0.89, respectively. It can be applied across various contexts, such as diagnosis of developmental conditions, clinical and neuropsychological evaluations, psychoeducational assessment of learning difficulties and special education needs, to determine the severity of intellectual disability and identify and qualify students for specialized educational programs (Roid et al. 2017a, 2017b). Critically for the present study, the SB5 includes no dedicated speeded subtests and provides no Processing Speed index; scoring is accuracy-based rather than time-weighted. Any administration time limits are procedural (standardization) and are not used to compute performance. Consequently, our SB5-based characterization of working memory and verbal/non-verbal reasoning is not confounded by speeded performance. Where the manuscript refers to “speed,” it pertains to the broader literature on dyslexia rather than to outcomes derived from the SB5. This choice reduces the risk that time pressure artificially depresses scores in participants with dyslexia, thereby improving the interpretability of profile-level differences.

### 2.3. Analytical Strategy

We employed LPA (Collins and Lanza 2010; Muthén 2002) to identify subgroups of participants with dyslexia characterized according to distinct patterns of intellectual functioning. The analysis was based on 10 standardized scores (scaled 1–19) derived from the SB5, which represented a comprehensive assessment of cognitive abilities across both verbal and non-verbal domains. This multivariate, person-centered approach was selected due to the substantial within-group heterogeneity often observed in individuals with dyslexia. LPA is a robust method for uncovering unobserved (latent) subgroups of individuals who share similar patterns of cognitive performance (e.g., Clark et al. 2024; Marawi et al. 2024).

To determine the optimal number of cognitive profiles, we estimated a series of LPA models that specified 2–6 latent profiles. Model selection was guided by a comprehensive fit indices and criteria: (a) the Akaike Information Criterion (AIC) and (b) Bayesian Information Criterion (BIC), where lower values indicate better model fit (Weller et al. 2020); (c) entropy, which ranged from 0–1, where higher values reflect further accurate classification (Weller et al. 2020); (d) Minimum Average Latent Class Probabilities (MALCP) for most likely class membership, which assesses the reliability of profile assignments (values ≥ 0.70 generally considered acceptable); (e) profile size, where classes that represented <5% of the sample are considered less interpretable or meaningful; and (f) REPLIC, which denotes whether the maximum log-likelihood value was replicated across multiple random starts. REPLIC reflects optimization stability: a value of 1 (yes) indicates that the highest log-likelihood was recovered at least twice and thus likely represents a global maximum; a value of 0 (no) indicates that the highest log-likelihood was not replicated, suggesting convergence to a local maximum and a potentially unstable solution. In the retained specification, class membership was modeled via class-specific means, whereas variances and covariances were held equal across classes (implemented in Mplus under %OVERALL%). Thus, the solution captures level differences (means) rather than structural (variances and covariances) differences.

Once the optimal solution was identified, profiles were characterized based on their mean standardized SB5 scores across the 10 domains. We conducted independent-samples *t*-tests with Welch’s correction (to consider unequal variances between groups) to assess between-profile differences in cognitive functioning, and calculated Cohen’s d effect sizes (Cohen 1988) to estimate their magnitude. Additionally, we utilized one-sample *t*-tests and corresponding effect sizes and compared mean SB5 scores in each group against the theoretical population mean (*M* = 10, *SD* = 3) to contextualize cognitive performance.

To explore potential demographic correlates of profile membership (RQ2), we examined whether age, gender, or parental education were associated with classification into the identified profiles. Chi-squared tests were conducted for gender and parental education (categorical variables) while *t*-tests assessed mean differences across profiles for age (continuous variable).

All analyses were conducted using Mplus version 8.11 (Muthen and Muthen 2017) and R version 4.5.1 (R Core Team 2025). The data and analysis code are available in an online repository (https://osf.io/mkp3a/?view_only=494c9ec6a8d54d8c8803e23d522805d1) (accessed on 12 September 2025) to enable open and transparent analyses.

## 3. Results

### LPA Results

LPA models that specified 2–6 profiles were evaluated via the AIC, BIC, entropy, profile size, and MALCP (see Table 2). Both AIC and BIC scores decreased as the number of profiles increased, which indicated incremental improvements in model fit. Entropy values ranged from 0.89–0.93, and the two-profile solution demonstrated the highest classification accuracy. However, high entropy alone did not guarantee an optimal or interpretable model when class sizes were extremely small. Compared to other solutions, the two-profile solution produced a more stable and interpretable distribution (5% vs. 95%), which reflected a clear cognitive contrast between the two subgroups. Conversely, solutions that specified 3–6 profiles resulted in highly unbalanced class distributions, and several classes dropped <5% of the sample and even <1% in the five- and six-profile solutions. Extremely small classes were typically considered unstable and of limited theoretical value. MALCP values decreased as model complexity increased (0.87–0.74), which reflected higher classification uncertainty in further complex models. Although the five- and six-profile models yielded slightly lower AIC and BIC values, they produced extremely small or unstable classes, which limited their interpretability. Therefore, the two-profile model best balanced statistical adequacy and theoretical clarity and distinguished a small subgroup with globally low cognitive functioning (Profile 1: Globally Low Cognitive Functioning) from a dominant subgroup with average cognitive abilities (Profile 2: Average Cognitive Functioning). This parsimonious two-profile solution was retained as optimal, consistent with both empirical evidence and theoretical framework of dyslexia heterogeneity.

**Figure 1:**
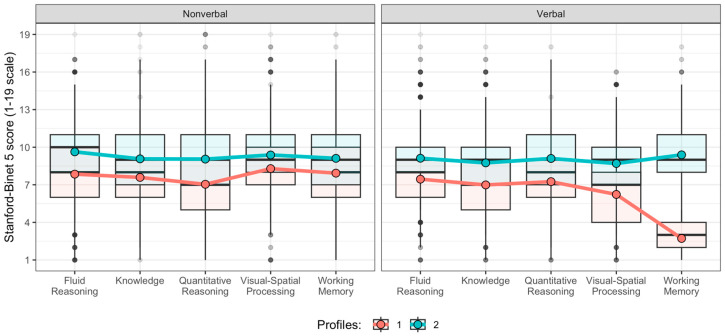
Latent Profiles of Cognitive Functioning Based on SB5 Standard Scores in Participants with Dyslexia.

Figure 1 illustrates the two identified profiles. Table 3 presents the descriptive statistics for all SB5 domains by profile, along with *t*-test results and Cohen’s d effect sizes. Independent-samples *t*-tests with Welch’s correction revealed statistically significant differences across all 10 SB5 domains between the profiles (all *p* < .01).

Profile 1: Globally Low Cognitive Functioning (*n* = 157; 5%) was characterized by markedly below-average performance across both verbal and non-verbal domains. Mean SB5 scores ranged from approximately 6.23–8.29, with the most pronounced deficits observed in Verbal Visual-Spatial Processing (*M* = 6.23), Verbal Knowledge (*M* = 6.99), and Quantitative Reasoning (*M* = 7.04–7.25). Notably, the mean score of Verbal Working Memory was only 2.72 (*SD* = 1.23), which was <3 *SD* below the population mean. This extreme result indicated a profound deficit in this cognitive domain. Compared with the general population norm (*M* = 10, *SD* = 3), all scores were significantly lower (*t* = −8.43–(−73.91), *p* < .01), and effect sizes ranged from *d* = −0.67–(−5.90), which supported this group’s classification as cognitively at-risk.

Profile 2: Average Cognitive Functioning (*n* = 3301; 95%) represented the majority and exhibited performance within the average range across all SB5 domains. Mean scores were generally close to the normative mean of 10 (range 8.70–9.63). Highest scores were observed in Non-verbal Fluid Reasoning (*M* = 9.63) and Verbal Working Memory (*M* = 9.39), which suggested relatively intact cognitive functioning. Compared with the theoretical population distribution, differences were statistically significant in all domains (*p* < .01); however, the small-to-moderate effect sizes (*d* = −0.14–(−0.50)) suggested slight but consistent underperformance relative to normative expectations.

Effect sizes for between-profile comparisons were substantial, ranging from *d* = −0.46–(−4.06), which confirmed a marked cognitive contrast between the two profiles. The largest between-group differences were observed for Verbal Working Memory (*d* = −4.06), Verbal Visual-Spatial Processing (*d* = −0.90), and Quantitative Reasoning (*d* = −0.73–(−0.70)), which highlighted the severity and breadth of cognitive difficulties in Profile 1 relative to Profile 2.

These findings supported the hypothesis that individuals with dyslexia were not cognitively homogeneous and differed meaningfully in the overall level and pattern of their cognitive functioning across SB5 domains.

Although profile estimation in LPA includes variances and covariances among the 10 SB5 domains, in the retained model, these parameters were constrained to be class-invariant (M1). Inspection of estimates confirmed that between-class differences primarily reflected mean-level distinctions, consistent with a solution that captures level differences (means) rather than structural differences (variances and covariances) in overall ability. To evaluate whether the retained solution reflected level differences (means) rather than structural differences (variances and covariances), we fitted two additional specifications. Model M2 allowed class-specific variances while keeping covariances equal across classes; Model M3 allowed full class-specific covariance matrices (variances and covariances). Across models, class sizes, entropy, and MALCP were monitored for stability, and fit was compared using AIC/BIC/SABIC values. Relative to M1 (class-specific means; common variances/covariances), M2 modestly improved information criteria (ΔAIC = −210.6; ΔBIC = −149.1; ΔSABIC = −180.9), but classification quality deteriorated sharply (entropy from 0.93 to 0.43; MALCP diagonal = 0.80/0.84; class proportions by most-likely membership: 0.20/0.80). M3 further lowered AIC scores (ΔAIC = −310.0) and slightly lowered SABIC (ΔSABIC = −146.6) but worsened BIC scores (ΔBIC = +28.1) and again showed weak classification (entropy = 0.42; MALCP diagonal = 0.82/0.83; proportions 0.25/0.75). Taken together, these checks support the interpretation that the profiles differ in level (means across SB5 subtests) rather than in structure (variances and covariances), and they justify retaining the more parsimonious and well-classified M1 despite mixed information-criterion signals.

A chi-squared test of independence was conducted to examine whether gender was associated with profile membership. Association between gender and cognitive profile was not statistically significant, χ^2^(1) = 1.87, *p* = .17. In Profiles 1 (Globally Low) and 2 (Average Cognitive Functioning), 69.4% and 62.7% of participants were male and 30.6% and 37.3% were female, respectively. These proportions indicated a slightly higher representation of males in the lower-functioning profile; however, the difference was not statistically meaningful.

Furthermore, another chi-squared test of independence was conducted to assess whether maternal education was related to profile membership. The association was statistically significant, χ^2^(3) = 34.10, *p* < .01. In Profile 1 (*n* = 120), 12.5%, 30.8%, 35.8%, and 20.8% had mothers with primary or lower secondary, vocational, secondary, and higher education, respectively. In Profile 2 (*n* = 2241), the corresponding proportions were 6.2%, 17.2%, 31.9%, and 44.7%, respectively. Hence, lower and higher levels of maternal education were more prevalent in the lower and average cognitive functioning group, respectively. A similar pattern was observed for paternal education, with a significant association between education level and profile membership (χ^2^(3) = 17.40, *p* < .01). In Profile 1 (*n* = 65), 9.2%, 52.3%, 26.2%, and 12.3% had fathers with primary or lower secondary, vocational, secondary, and higher education, respectively. In Profile 2 (*n* = 1134), the corresponding values were 7.3%, 30.1%, 33.8%, and 29.3%, respectively. Hence, vocational and higher education was substantially more common among fathers of those in the lower and average functioning group, respectively. This further supported the association between parental education and cognitive functioning profiles in children with dyslexia.

Finally, an independent-samples *t*-test with Welch’s correction was conducted to determine whether age differed according to profile. The analysis revealed a statistically significant difference, *t*(172) = −4.24, *p* < .01. On average, participants in Profile 1 were younger (*M* = 12.5 years, *SD* = 1.91) than those in Profile 2 (*M* = 13.2 years, *SD* = 1.94). The effect size was small (*d* = −0.34), which indicated a modest but reliable age difference between the groups. This suggested that younger participants were slightly more likely to be classified into the lower-functioning profile.

## 4. Discussion

This study identified two distinct subgroups of children with dyslexia based on the full SB5 profile and LPA: a predominant subgroup (95%) with an average intellectual profile across SB5 domains and a small subgroup (5%) with globally depressed intellectual scores, who demonstrated a strikingly severe deficit in verbal working memory (*M* = 2.72), which corresponded to >3 *SD* below the theoretical norm. This finding was both statistically and clinically significant and represented the primary axis of differentiation between intellectual profiles. From the perspective of SLD heterogeneity, this pattern suggested that subgrouping in dyslexia may largely reflect a severity gradient within intellectual functioning, rather than qualitatively distinct categorical subtypes.

The robust association between verbal working memory (WM) and reading difficulties has been a replicated finding. Meta-analyses confirmed medium-to-large impairments in verbal WM among children with reading disabilities (Jerman and Lee Swanson 2005; Kudo et al. 2015). Importantly, our study demonstrated that such deficits emerged within the structure of intelligence itself, rather than only in broader cognitive domains. LPA research similarly identified distinct WM subgroups among children with dyslexia, which confirmed the centrality of WM as a differentiating intellectual factor (Gray et al. 2019). Therefore, the extremely low verbal WM scores in our minority subgroup constituted a genuine clinical marker within their intellectual profile, not a methodological artifact.

Conversely, the dominant class, characterized by average intellectual functioning across SB5 domains, relatively preserved fluid reasoning, and only mildly reduced verbal WM, resembled profiles observed in Wechsler batteries (General Ability Index > Cognitive Proficiency Index); WM and processing speed emerged as relative intellectual weaknesses although reasoning abilities were intact (Mather and Schneider 2023). This supported the view that examining profiles of intelligence provided a further nuanced understanding of dyslexia compared to reliance on a single global IQ score.

Our finding of two classes differentiated primarily by level of intellectual functioning, rather than by structural differences (variances and covariances), supports the dimensional perspective of intelligence in dyslexia. Consistent with this view, our sensitivity analyses showed that allowing class-specific variances and covariances did not yield a clearly superior and stable solution, reinforcing that the subgroup distinction reflects level (means) rather than structural (variances and covariances) differences. This aligned with contemporary frameworks that shifted from discrepancy-based IQ-achievement models, long criticized for their lack of validity (Fletcher and Miciak 2024; Stuebing et al. 2002). Previous LPA studies that used reading-specific measures uncovered multiple profiles even in early literacy development (Gray et al. 2019). Hence, whether dyslexia appears dimensional or categorical may depend on the level of analysis: when intelligence is the analytic frame, subgrouping reflects the severity of intellectual functioning; when specific reading or language processes are measured, further fine-grained subtypes may emerge. From a practical perspective, the two-profile solution suggests that intellectual assessment is most informative for identifying quantitative differences in overall functioning rather than qualitatively distinct forms of dyslexia. Thus, while subgroup membership should not be used to assign children to rigid categories, cognitive assessment may help to pinpoint relative weaknesses (e.g., in working memory, processing efficiency, or verbal reasoning) that interact with reading difficulties. These findings underscore the importance of tailoring interventions to individual cognitive vulnerabilities while maintaining evidence-based reading instruction as the foundation of treatment.

The Pattern of Strengths and Weaknesses (PSW) approach, which emphasizes the diagnostic relevance of uneven cognitive profiles, recognized as dyslexia characteristic, is a related framework (Hale et al. 2010; Mather and Schneider 2023). Unlike the traditional IQ-achievement discrepancy, PSW compares intact and impaired cognitive abilities to capture the unexpectedness of SLD (Kavale and Forness 2000; Schultz et al. 2012). Our finding of a subgroup with preserved reasoning but severely impaired verbal WM supports this logic; hence, LPA applied to a comprehensive intelligence battery can be viewed as a data-driven operationalization of PSW. Although PSW remains debated regarding psychometric robustness and its impact on instructional planning (Elliott and Resing 2015; Maki et al. 2022), our results illustrate how person-centered statistical methods can strengthen and objectify the identification of intra-individual patterns. Thus, use of the SB5, which covers five factors of intelligence, captured the internal architecture of intellectual functioning in dyslexia. This perspective emphasizes that dyslexia is not only about reading skills but also the broader profile of intellectual strengths and weaknesses, especially the critical role of verbal WM.

The observed association between lower parental education and membership in the low-intellectual-functioning class highlights that socio-educational context shapes the development of intellectual abilities, not just general cognitive outcomes. Recent evidence has demonstrated that socio-economic status (SES) moderated both the level and mechanism of reading difficulties; children from higher and lower SES often exhibited phonological deficits and rapid naming deficits, respectively, supported by distinct neural profiles (Romeo et al. 2022). These findings underline the importance of considering SES in language and reading outcomes and development of specific intellectual factors, such as verbal WM (Taylor et al. 2023).

The finding that the low-functioning subgroup was younger may reflect both developmental catch-up processes and earlier identification of students with globally compromised intellectual profiles. However, longitudinal evidence suggests that children with reading difficulties do not spontaneously catch up over time (Shaywitz et al. 1999). Furthermore, early cognitive or preliteracy profiles remain remarkably stable and predictive of later reading outcomes (Ozernov-Palchik et al. 2017). Therefore, longitudinal research should determine whether SB5-based intellectual subgroups persist or whether targeted educational interventions can alter their trajectories.

Our findings have several implications for the assessment and support of students with dyslexia. First, consistent with Fletcher and Miciak (2024), profile-based analysis of intelligence, such as detailed examination of the SB5 subtest patterns, should replace reliance on the traditional IQ-achievement discrepancy model, which lacks empirical validity in SLD identification. Second, although targeted interventions focused on verbal WM may seem promising, WM training only produces short-term near-transfer effects and does not generalize to broader academic skills, such as reading comprehension (Melby-Lervåg and Hulme 2013; Melby-Lervåg et al. 2016). Finally, our results underscore the importance of SES-sensitive support: children from lower SES backgrounds often exhibit linguistic, reading, and executive function disadvantages, which suggests the value of enriched home literacy environments and family engagement strategies (Romeo et al. 2022; Taylor et al. 2023).

This study has several limitations. First, the factorial structure and reliability of the SB5 subtests have been extensively validated in the Polish adaptation (Roid et al. 2017a). Given that our LPA was conducted on standardized subtest scores, we did not re-test the CHC-based structure within the current dataset. Future research could extend these analyses using latent-variable approaches to account for measurement error at the item level. Second, although the SB5 examined the profile of intelligence, it was not optimized to identify subtle, reading-specific subtypes (e.g., phonological vs. RAN-driven). Future studies should integrate the SB5 with specialized literacy and neuroimaging measures to link intellectual profiles with reading-specific mechanisms (Gray et al. 2019). Third, the clinical sample and incomplete SES data limit generalizability. Due to the high proportion of missing data for parental education, particularly paternal education, associations between socioeconomic indicators and cognitive profiles should be interpreted with caution. Although the observed patterns were consistent and statistically significant, they warrant replication using datasets with more complete SES information. Fourth, the cross-sectional design precludes conclusions regarding developmental stability; further longitudinal research is required. Finally, although our findings support a dimensional view of intelligence in dyslexia, this does not preclude the existence of distinct mechanistic subgroups when examined through other domains (Fletcher and Miciak 2024).

## 5. Conclusions

Identification of two intellectual profiles in dyslexia, with the key distinction anchored in verbal working memory, supports a dimensional view of heterogeneity. Our study focused on the structure of intelligence, provided evidence against IQ-achievement discrepancy criteria, and underscores the value of profile-based interpretation of intelligence tests. These findings have direct implications for diagnosis, classification, and intervention, and emphasize that dyslexia should be understood not only as reading deficits but also through the lens of intellectual architecture.

## Figures and Tables

**Table 1:** Participants’ Demographic Characteristics.

Variable	*N*	%
*Gender*		
Female	1245	36
Male	2213	64
*Place of Residence*		
Countryside	1007	29
City	2443	71
Missing data	8	<1
*Mother’s Education*		
Primary or Lower Secondary	155	5
Vocational	422	12
Secondary	758	22
Graduate and Postgraduate	1026	30
Missing data	1097	31
*Father’s Education*		
Primary or Lower Secondary	89	3
Vocational	375	11
Secondary	400	12
Graduate and Postgraduate	340	9
Missing data	2254	65

**Table 2:** Model Fit Indices, Entropy, and Profile Sizes for LPA of SB5 Scores.

Model	AIC	BIC	Final Class Proportions for the Latent Classes Based on Their Most Likely Latent Class Membership						Entropy	MALCP	REPLIC
			p1	p2	p3	p4	p5	p6			
2 profiles	153,984	154,451	.05	.95					0.93	0.87	Yes
3 profiles	153,892	154,428	.04	.94	.02				0.92	0.79	Yes
4 profiles	153,816	154,418	.01	.04	.03	.92			0.91	0.82	Yes
5 profiles	153,743	154,413	.04	.01	.92	.03	<.01		0.93	0.83	No
6 profiles	153,698	154,436	.01	<.01	.04	.89	.03	.03	0.89	0.74	Yes

Note. AIC = Akaike Information Criterion; BIC = Bayesian Information Criterion; MALCP = minimum average latent class probabilities for most likely latent class membership; REPLIC = the best loglikelihood value has been replicated.

**Table 3:** Descriptive Statistics and Between-Profile Comparisons for SB5 Cognitive Domains.

Variable	Profile 1	Profile 2	Profile 1 vs. Profile 2		Profile 1 vs. Population Norm		Profile 2 vs. Population Norm	
	*M* ± *SD*	*M* ± *SD*	*t*	*d*	*t*	*d*	*t*	*d*
Non-verbal								
Fluid Reasoning	7.85 ± 2.87	9.63 ± 2.71	−7.60 **	−0.64	−9.39 **	−0.75	−7.90 **	−0.14
Knowledge	7.59 ± 2.91	9.07 ± 2.78	−6.22 **	−0.52	−10.36 **	−0.83	−19.25 **	−0.34
Quantitative Reasoning	7.04 ± 2.55	9.05 ± 2.76	−8.98 **	−0.73	−13.56 **	−1.08	−19.79 **	−0.34
Visual-Spatial Processing	8.29 ± 2.55	9.38 ± 2.24	−5.28 **	−0.46	−8.43 **	−0.67	−15.93 **	−0.28
Working Memory	7.93 ± 2.61	9.11 ± 2.46	−5.52 **	−0.46	−9.92 **	−0.79	−20.86 **	−0.36
Verbal								
Fluid Reasoning	7.45 ± 2.81	9.12 ± 2.33	−7.34 **	−0.65	−11.39 **	−0.91	−21.71 **	−0.38
Knowledge	6.99 ± 2.98	8.75 ± 2.56	−7.28 **	−0.63	−12.67 **	−1.01	−28.11 **	−0.49
Quantitative Reasoning	7.25 ± 2.73	9.10 ± 2.57	−8.34 **	−0.70	−12.65 **	−1.01	−20.14 **	−0.35
Visual-Spatial Processing	6.23 ± 2.87	8.70 ± 2.59	−10.57 **	−0.90	−16.47 **	−1.31	−28.90 **	−0.50
Working Memory	2.72 ± 1.23	9.39 ± 1.97	−63.97 **	−4.06	−73.91 **	−5.90	−17.68 **	−0.31

Note. *N*_Profile1_ = 157; *N*_Profile2_ = 3301; ** *p* < .01; Population Norm = theoretical (expected) score distribution in the general population (*M* = 10).

## Data Availability

The original data presented in the study are openly available in Open Science Framework (OSF) at https://osf.io/mkp3a/?view_only=494c9ec6a8d54d8c8803e23d522805d1 (accessed on 12 September 2025).

## Abbreviations

The following abbreviations are used in this manuscript:

LPA	Latent Profile Analysis
SB5	Stanford-Binet Intelligence Scales, Fifth Edition
SES	Socio-economic Status
AIC	Akaike Information Criterion
BIC	Bayesian Information Criterion
MALCP	Minimum Average Latent Class Probabilities
SLD	Specific Learning Disorder
PSW	Pattern of Strengths and Weaknesses
WM	Working Memory
